# Cpd-1 Null Mice Display a Subtle Neurological Phenotype

**DOI:** 10.1371/journal.pone.0012649

**Published:** 2010-09-09

**Authors:** Rupinder K. Kular, Rocky G. Gogliotti, Puneet Opal

**Affiliations:** 1 Davee Department of Neurology, and Department of Cell and Molecular Biology, Northwestern University Feinberg School of Medicine, Chicago, Illinois, United States of America; 2 Integrated Graduate Program, Northwestern University Feinberg School of Medicine, Chicago, Illinois, United States of America; University of Florida, United States of America

## Abstract

**Background:**

CPD1 (also known as ANP32-E) belongs to a family of evolutionarily conserved acidic proteins with leucine rich repeats implicated in a variety of cellular processes regulating gene expression, vesicular trafficking, intracellular signaling and apoptosis. Because of its spatiotemporal expression pattern, CPD1 has been proposed to play an important role in brain morphogenesis and synaptic development.

**Methodology/Principal Findings:**

We have generated CPD1 knock-out mice that we have subsequently characterized. These mice are viable and fertile. However, they display a subtle neurological clasping phenotype and mild motor deficits.

**Conclusions/Significance:**

CPD1 is not essential for normal development; however, it appears to play a role in the regulation of fine motor functions. The minimal phenotype suggests compensatory biological mechanisms.

## Introduction

Development of the nervous system is a well-coordinated process that relies on the ability of neurons to differentiate, migrate, extend neurites and form synapses. How each of these processes is brought about is still obscure. To address this shortcoming, we have taken the approach of exploring the role of candidate developmental orchestrators—specifically, proteins whose expression patterns mirror key developmental time-lines, and are also likely to play important cellular functions likely pivotal for neuronal development. To this end, we have focused our efforts on members of the conserved family of acidic nuclear proteins (ANPs). In previous work, we have explored the role of the leucine rich acidic nuclear protein (LANP; also named, pp32, I1PP2a, PHAPI, mapmodulin; ANP32-A) [1]–[4]. In the present manuscript, we focus on its closely related family member, Cerebellar Postnatal Developmental Protein-1 (CPD1, also named LANP-like, LANPL, ANP32-E) [5]. LANP and CPD1 were originally identified as candidate determinants of neuronal architecture based on their tightly regulated expression pattern during development. LANP (mouse LANP: 247 amino acids; NCBI Reference Sequence: NP_033802.2) was the first to be discovered, identified on the basis of a proteomic screen for developmentally regulated proteins in the rat brain peaking in the first few weeks of life [6]. CPD1 (260 amino acids; NCBI Reference Sequence: NP_075699.3) was subsequently discovered using similar high-throughput methods; although in this case the technique of differential display was used to identify messenger RNAs that increase in the immediate postnatal period [7].

LANP—the better studied of the two— plays an important role in regulating neurite outgrowth and neurodegeneration, likely by its combined ability to regulate gene expression and microtubule dynamics [8](Ulitzur et al., 1997)[4], [9]. Both proteins have been described as inhibitors of protein phosphatase 2A activity [10], [11], with CPD1 thought to modulate phosphatase activity at synapses during synaptogenesis [11]. Besides these properties, LANP and other ANPs have also been proposed to play a role in the regulation of pathways involved in signaling [12], apoptosis [13], [14] and RNA transport and stability [15]. In addition to their role in the nervous system, there is a substantial body of work that suggests that the ANP family of proteins plays a significant role in modulating tumorigenesis with prognostic implications [16], [17], [18]. Despite these wide-ranging properties LANP null mice have no discernable behavioral phenotype suggesting functional compensation by other ANP family members[3].

Here we report the generation and characterization of CPD-1 null mice. Because of our interest in its neuronal role, we have studied these mice from a neurobehavioral perspective. Apart from abnormal movements such as clasping, and mild impairments in gait, these mice are virtually indistinguishable from wild-type mice. Finally, we have mated CPD1 null mice with LANP null mice to discover that mice lacking both LANP and CPD1 are also viable and fertile. We propose that the functions of these proteins are usurped by other member(s) of the ANP class of proteins that will likely be uncovered by yet other genetic deletion experiments.

## Results

### Generation of CPD1 null mice

To generate CPD1 null mice we took advantage of recent developments in high-throughput insertional mutagenesis of mouse embryonic stem cells (ES cells) to generate knock-out mice. These ES cells are available to investigators from the International Gene Trap Consortium (IGTC). An ES clone (RRK001) with the CPD1 locus targeted was used for blastocyst injections to generate chimeras. Germline transmission was confirmed by genotyping for the β-Geo insertion cassette. Heterozygous CPD1 targeted mice were mated to generate CPD1 null mice (see Figure S1 for further details). Mice lacking CPD1 are viable and fertile. Breeding of CPD1 heterozygous mice resulted in roughly Mendelian ratios of progeny (Table 1).

**Table 1:** Breeding of CPD1 heterozygous mice resulted in roughly Mendelian ratios of progeny.

	CPD1 +/+	CPD1 +/−	CPD1 −/−
**Expected %**	25%	50%	25%
**Expected number**	23.5	47	23.5
**Obtained number**	20	46	28
**Obtained %**	21.27%	48.9%	29.8%

**Figure 1:**
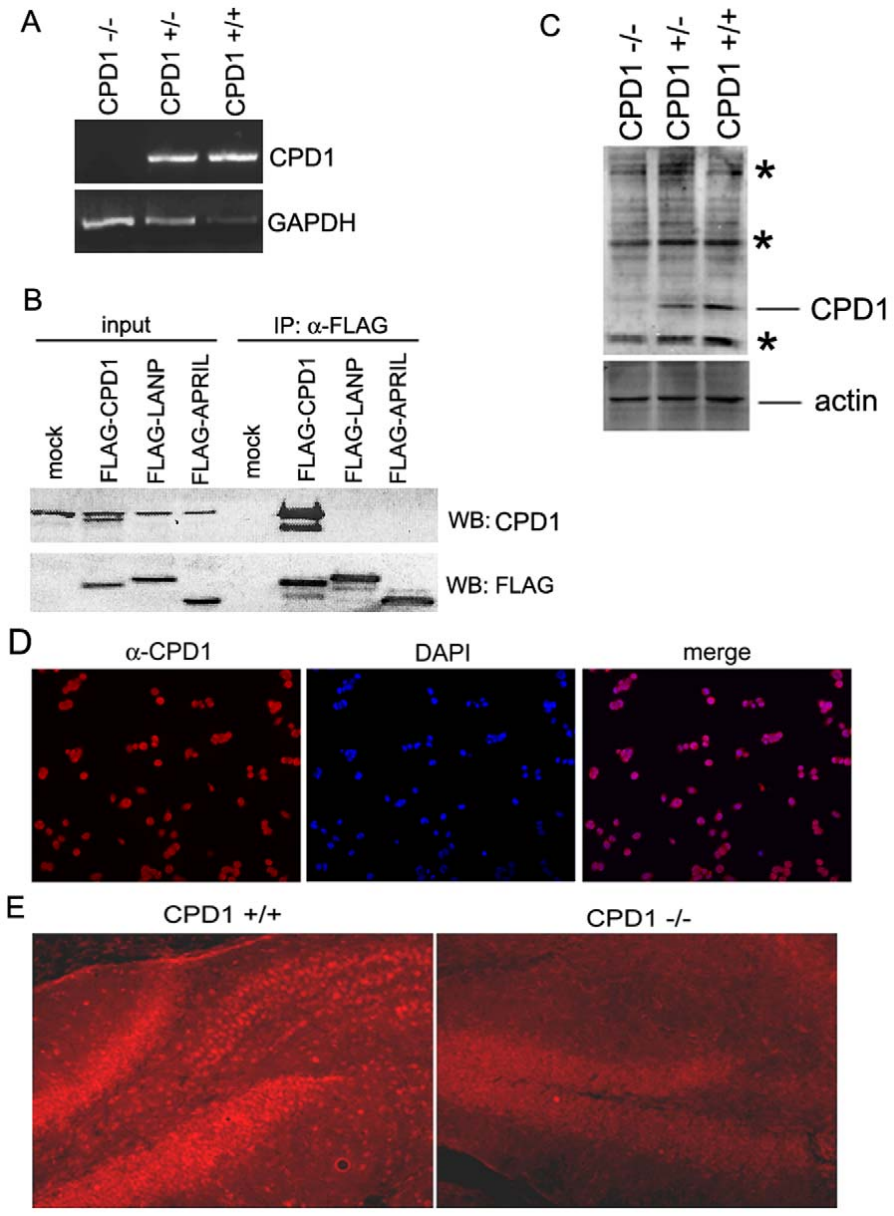
Generation of CPD1 null mice. A. Loss of CPD1 message in CPD1 null mice. RNA extracted from brains of CPD1 +/+, CPD1 +/−, and CPD−/− mice show loss of CPD1 transcripts by RT-PCR analysis. GAPDH is used as an internal control. Reduction in CPD1 transcript level is evident in CPD1 +/− mice. B. Antibody to CPD1 does not cross-react against ANP homologues. FLAG-tagged versions of CPD1, LANP and APRIL were transfected into cells and immunoprecipitaed with anti-FLAG antibody. The antibody to CPD1 specifically recognizes CPD1 and not the other ANP homologues. Western blot with FLAG demonstrates the transfection and pull-down of the relevant proteins. C. CPD1 null mice do not express CPD1 protein. Protein extracted from brains of CPD1 +/+, CPD1 +/−, and CPD−/− mice show levels of CPD1 protein by western blot analysis. Actin is used as loading control. Asterisks denote non-specific bands. D. Expression of endogenous CPD1. Immunostaining of neuro2a cells with α-CPD1 antibody displays localization of CPD1 protein. DAPI staining marks the nucleus. E. Immunostaining on brains dissected from CPD1 +/+ and CPD1 −/− mice shows reduced CPD1 staining in CPD1 null mice.

To confirm that these mice lack CPD1 we tested for the presence of CPD1 messenger RNA by Reverse-Transcriptase PCR (RT-PCR) on RNA extracted from brains of CPD1+/+, CPD+/− and CPD1−/− mice. Loss of CPD1 transcript is evident from the RT-PCR analysis (Figure 1A). (Similar results were also obtained from RNA derived from other organs such as kidney; data not shown).

There have been several antibodies purportedly synthesized against CPD1. We tested these antibodies against CPD1 by transfecting cells with FLAG-tagged CPD1 along with homologous FLAG-tagged ANP family members: LANP and APRIL (A Protein Rich in Leucines, aka PAL-31 and Anp32b). We then performed immunoprecipitations with FLAG antibody followed by western blots. We identified that a chicken antibody made by GenWay Biotech (San Diego, CA) recognizes the CPD1-FLAG protein. More importantly, this antibody does not cross-react against two ANP homologues (LANP and APRIL) (Figure 1B). Next, using this antibody we wished to determine loss of CPD1 protein in CPD1 null mice by western blotting. Although, this antibody recognizes several non-specific bands, we demonstrate that a protein band of the estimated molecular weight seen in wild-type mice disappears in CPD1 null mice (Figure 1C). (Heterozygous mice demonstrate approximately a 50% reduction in the level of CPD1 protein). To further, explore the specificity of this antibody, we depleted CPD1 in PC12 (Rat Pheochromocytoma cells) using RNA interference (Text S1). Depletion of CPD1 is evident from the western blot analysis (more than 75% knockdown; Figure S2A) and immunofluorescence microscopy (Figure S2B).

Using this antibody for immunofluorescence we find that CPD1 displays predominantly nuclear staining with some cytoplasmic staining. This is the case both for cells *in vitro* (neuro2a cells are shown as an example; Figure 1D) and *in vivo* (a hippocampal slice is shown as an example; Figure 1E). In the course of these experiments we could not find any alterations in histological morphology or layering of neuronal populations (data not shown).

### Characterization of CPD1 null mice

Because of the described role of CPD1 in the nervous system, we focused our subsequent efforts on characterizing the neuronal phenotype of CPD1 null mice. Moreover, because CPD1 has been described as a protein involved in cerebellar morphogenesis, we particularly focused on evaluating the motor skills of CPD1 null mice.

**Figure 2:**
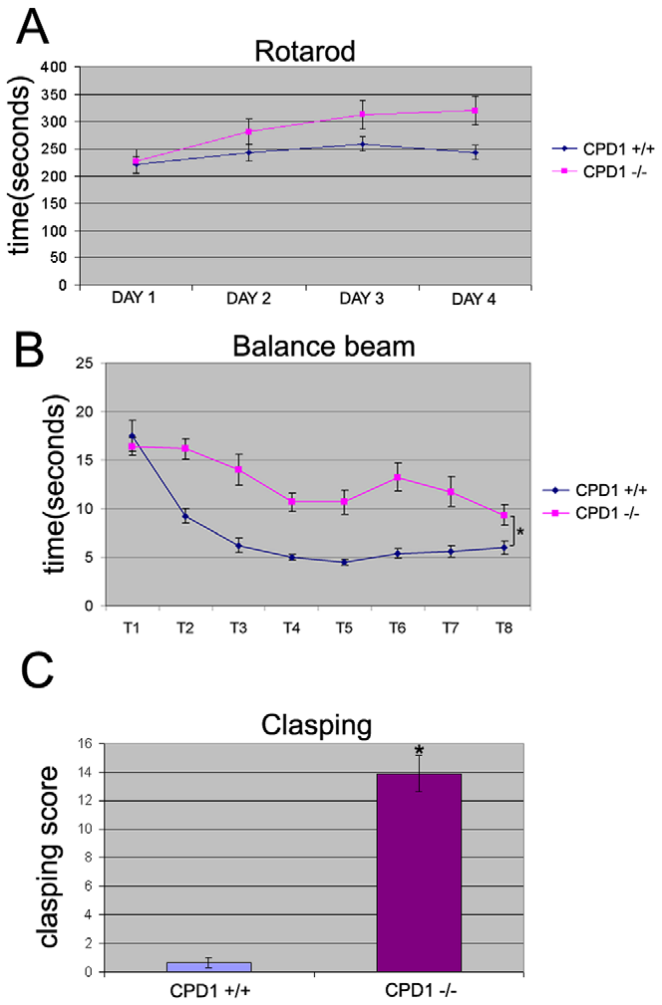
Analysis of motor deficits in CPD1 null mice. A. Accelerating rotarod analysis: 4 month old CPD1 +/+ (n = 6) and CPD1 −/− (n = 12) were tested for a fall from a rotating rod. Average of 4 trails on each day is plotted. Error bars = SEM; p = 0.067, suggesting no significant changes. B. Dowel test: 4 month old CPD1 null mice require more time to traverse a balance beam. CPD1 +/+ (n = 6) and CPD1−/− (n = 12) were made to traverse a beam placed on two platforms for 8 trials on 8 days. CPD1−/− mice took longer time to traverse the beam (p<0.005). C. CPD1 null mice display clasping behavior. CPD1 +/+ (n = 3) and CPD1 −/− (n = 16) were held from their tails and tested for clasping of the limbs. CPD1 null mice clasp their limbs more times and for longer duration than age matched control (p<0.005).

To assess motor learning we tested CPD1 null mice on the accelerating rotarod test. This is an excellent screening test to discern impairments in locomotion and cerebellar motor learning. Mice impaired in motor function typically fall off the rotarod and do not improve with consecutive trials (4 trials per day over 4 consecutive days). As shown in Figure2A, CPD1 null mice do not display any motor deficits in this assay when compared to age-matched controls.

We next tested gait in a more challenging situation. Specifically, mice were tested in their ability to traverse a narrow balance beam (a wooden dowel suspended at a height). In this test, we found that CPD1 null mice perform worse than their littermates taking a longer time to traverse the dowel between two platforms (Figure 2B). Consistent with these motor deficits, we also observed that CPD1 null mice tend to clasp their limbs (pull their limbs towards the abdomen rather than extend them outwards which is the normal response) while suspended from their tail (Movie S1 and Movie S2). This has been quantified in Figure 2C.

**Figure 3:**
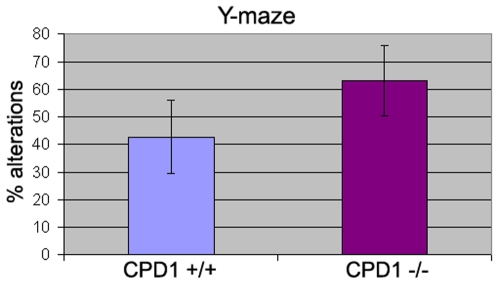
CPD1 null mice do not depict any memory related behavioral changes tested by Y maze. 4 month old CPD1 +/+ (n = 3) and CPD1−/− (n = 8) mice were tested on Y maze for spontaneous alterations to enter arms of Y maze. Mice were tested for 5 minutes or 22 entries (whichever came first) in arms of a Y maze. No significant difference in % of alterations were observed (p>0.05).

Finally, because we find that CPD1 expression is also quite abundant in hippocampal neurons, we tested CPD1 null for spatial memory using the Y maze. However, no significant differences in behavior were observed in CPD1 null mice (Figure 3). In sum, CPD1 null mice display only subtle motor phenotypes.

**Figure 4:**
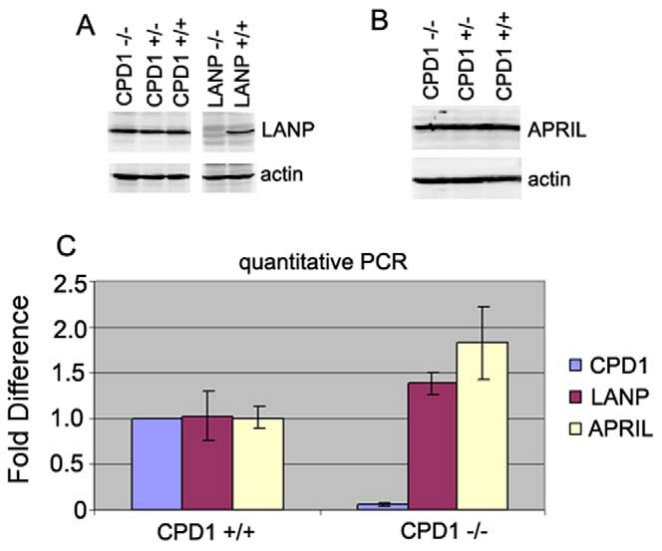
Expression levels of LANP and APRIL do not alter in CPD1 null mice. A. Brain lysates from CPD1 +/+, CPD1 +/−, and CPD1 −/− mice were subjected to western blot analysis with an anti-LANP antibody to check for protein levels of LANP. Lysate from LANP null mice served as control with actin as loading control. B. Brain lysates from CPD1 +/+, CPD1 +/−, and CPD1 −/− mice were subjected to western blot analysis to check for protein levels of APRIL with an APRIL antibody. C. mRNA expression analysis of CPD-1, LANP and APRIL was performed using quantitative PCR. GAPDH served as an internal control and the fold difference is relative to expression in wild type mice.

Because CPD1 is closely related to two other acidic nuclear proteins LANP (Anp32a) and APRIL (Anp32b), we next wished to determine the levels of LANP and APRIL in CPD1 null mice to see if there might be any compensatory upregulation. Brain lysates from CPD1 null mice were subjected to western blot analysis. No significant change in protein levels of LANP and APRIL was observed (Figure 4A and B). There was a trend in increase in the level of RNA transcripts of LANP and APRIL; however, these differences were not statistically significant (p>0.05) (Figure 4C).

Previously, we have generated LANP null mice that also like CPD1 null mice are viable and fertile without significant deficits in home cage behavior and several behavioral assays [3]. To test whether depleting two ANP family members might uncover more obvious deficits we crossed the LANP null and CPD null mouse lines. To date these mice are viable and fertile and do not display major behavioral phenotypes on home cage behavior. Viability of double knockout mice suggests that other ANP homologues are likely to compensate for loss of these proteins.

## Materials and Methods

### Ethics Statement

All protocols involving the use of animals were in compliance with the National Institutes of Health's Guide for the Care and Use of Laboratory Animals and the Northwestern University Institutional Animal Care and Use Committee (Protocol 2009–1751 and protocol 200801411v1). These protocols are based on our proposals to study the role of ANP proteins in development and neurodegeneration—including this current study. Northwestern University has an Animal Welfare Assurance on file with the Office of Laboratory Animal Welfare (A3283-01).

### Generation of mouse models

#### Generation of CPD1 null mice

Embryonic stem (ES) cells derived from 129P2/OlaHsd mouse strain with the CPD1 targeted with a gene trap were obtained from BayGenomics (MMRRC BayGenomics, UC Davis) [19]. The gene trap vector (pGTLxf) contains a splice acceptor sequence upstream of a β-Geo reporter gene (β-galactosidase fused to a neomycin selection cassette). 5′ RACE (rapid amplification of cDNA ends) analysis suggested that the gene trap had inserted in the intron between exon 3 and 4 of the mouse CPD1 gene. ES cells were then injected into C57BL/6 blastocysts to generate chimeras (at the Murine Targeted Genomics Laboratory; University of California, Davis). Chimeras were mated to C57/BL6 mice (Jackson Laboratory, Bar Harbor). Germ line transmission was determined taking advantage of coat color of progeny and subsequent genotyping (see supplementary information; Text S1). Primers to genotype the CPD1 mice were generated by testing various primers designed from the mouse genome sequence on the Ensembl database (Mouse Genome Sequencing Consortium).

#### Generation of LANP null mice

The generation of LANP null mice has been previously described [3].

### RT-PCR analysis

RNA was isolated from brains of adult mice (6 weeks of age) of the described genotypes using Trizol (invitrogen). cDNA was synthesized using Transcriptor first strand synthesis kit (Roche), and quantitative PCR was performed as described [9]using 2X PCR mix (Fermentas) employing following primers: CPD1 Fwd: ATCGAAGGCCTGAATGACAC; CPD1 Rev: GTCATCCTCCTCCTCCGAGT. LANP Fwd: TGGAGATGGACAAACGGATT; LANP Rev: GGCAGGAGCTTGAACACG. APRIL Fwd: TAGCAGAAGAACTTCCAAGC; APRIL Rev: CATAGCCATCCAGATAGGAC. GAPDH Fwd:ACAACTTTGGCATTGTGGAA GAPDH Rev: GATGCAGGGATGATGTTCTG.

### Cell lines and cell culture

Mouse neuroblastoma neuro2a (N2A) cells (ATCC) were grown in Dulbecco's modified Eagle's medium containing 10% FBS, sodium pyruvate, penicillin/streptomycin, and non-essential aminoacids. PC12 (Rat Pheochromocytoma cells) were grown in Dulbecco's modified Eagle's medium containing 8% FBS, 8% horse serum, penicillin/streptomycin, and non-essential aminoacids.

### Immunoprecipitation

HeLa cells were plated at the cell density of 2×10^6^ cells/10 cm plate. 24 h later the cells were transfected with FLAG-CPD1, FLAG-LANP [4]and FLAG-APRIL (gift from Dr. Joan Steitz) [15] using lipofectamine (Invitrogen) as the transfection reagent. 48 h post transfection, the cells were lysed in lysis buffer (5 mM EDTA and 0.5% Triton X-100 in PBS). Lysates were cleared by centrifuging at high speed, incubated with 2 µg of anti-FLAG antibody, clone m2 (Sigma) overnight, and immunocomplexes were pulled down by protein A/G agarose beads (Clontech). Immunoprecipitates were washed five times with PBS and the beads were boiled with 3X SDS sample buffer before loading onto a denaturing gel. The membrane was subjected to western blot analysis employing antibodies against the FLAG epitope (M2 antibody, Sigma), and CPD1 (# 15-288-21207 GenWay Biotech San Diego).

### Western blot analysis on mouse tissue lysates

Tissue lysates derived from euthanized mice were homogenized in lysis buffer (100 mM Tris pH 6.8, 25 mM dTT, 2% SDS, 5X protease inhibitor mix (complete protease inhibitor tablets; Roche)) using 2 ml of buffer for every 250 mg of tissue. 40 µg of protein was separated onto a denaturing SDS gel and western blotted using antibodies against CPD1 (# 15-288-21207 GenWay Biotech San Diego), actin (# A5441 Sigma), LANP (3118: [4]) and APRIL (aka PAL31, Anp32b [20])).

### Immunofluorescence

#### Immunofluorescence on tissue cultured cells

N2A cells were plated on coverslips at cell density of 250,000 cells/coverslip. 24 h later the cells were fixed with 4% formaldehyde and stained with primary antibodies (anti-CPD1; GenWay) and fluorescently labeled secondary antibody (Jackson Immunochemicals). Microscopy was performed using a Zeiss Axiovert microscope. Images were processed with Adobe Photoshop 7.0 software.

#### Immunofluorescence on fixed brain tissue

Brains from euthanized mice were fixed in 4% paraformaldehyde overnight, followed by incubation in 30% sucrose for 24 hrs. Fixed brains were then embedded in OCT (Tissue Tek), and sections of 40 µm thickness were obtained. Sections were permeabilized with Phosphate buffered saline with Triton X (0.3% Triton X -100 in PBS) for 10 minutes, blocked in 5% normal donkey serum (Jackson Immunochemical) and incubated with CPD1 antibody (GenWay) overnight. The samples were washed in Tris buffered saline with Tween 20 (Tris pH 7.5, 145.5 mM NaCl and 0.1% Tween 20), and incubated with fluorescently labeled secondary antibody (Jackson Immunochemical). Upon washes in TBST, the sections were mounted in mounting media containing DAPI (Vectashield) and visualized with Zeiss Axiovert Microscope.

### Behavioral Assays

#### (i) Rotating rod analysis

Three month old mice were placed on a rotating-rod apparatus that accelerates linearly from 4 to 40 rpm over five minutes of the run, followed by rotating at 40rpm for the next five minutes. Mice were tested in four trials on four consecutive days. The amount of time a mouse stayed on the rotating rod was plotted to a maximum time of 10 minutes, the duration of each trial. Two episodes of holding as the rod rotated through 360 degrees were also scored as a fall. The average for four trials for each day was plotted.

#### (ii) Balance Beam

Mice were tested for motor coordination using a balance beam [21]. Briefly, a cylindrical beam (11 mm in diameter, and 41 cm in length) was placed horizontally on two platforms, 50 cm above a table. A bright light illuminated the start platform, and a darkened enclosed escape box (20×20×20 cm) was situated at the other end of the table. The time to traverse each beam was recorded for each trial with a 20s maximum cutoff (falls were scored as 20s). The mice were trained for the first three trials on three consecutive days (T1-3), and then tested on five consecutive days (T4-8).

#### (iii) Limb clasping analysis

Mice were tested for limb clasping behavior by holding the mice from tail and suspending for 30 seconds. Mice were scored positive for clasping if they held the limb towards their belly, rather than extended outward. Arbitrary scores were given as follows: <5s clasp = 1; >5s and <10s = 5; >10s = 10. Scores were calculated and an average from each genotype was plotted.

#### (iv) Y maze

Mice were tested for spontaneous alterations using Y maze. Mice were placed in the “start” arm of the Y maze and left to roam freely until it has made 22 entries or until 5 minutes have elapsed. Three consecutive choices in three different arms were considered an alteration. The average of percentage alterations was plotted.

### Statistics

All statistical analyses were performed using GraphPad (Prism) using two-tailed t test. Paired t test were used for rotating rod assay and balance beam where equal number of data points representing trials for each experimental genotype were compared, while unpaired t test was used for clasping and Y maze tasks where two groups containing unequal number of mice represent the experimental groups.

## Discussion

The ANP-family of proteins multitask in a variety of functions in both the cytoplasm and the nucleus [5]. CPD1 is a relatively understudied member of this family. In this study, we have generated and characterized mice lacking CPD1 that we demonstrate are viable and fertile.

Using an antibody validated with the help of our CPD1 knock-out mice, we show that in tissue lysates derived from wild-type mice CPD1 that runs at 34 kDa. Moreover, at a subcellular level, CPD1 is present predominantly in the nucleus with some cytoplasmic staining. The beta-galactosidase mutagenesis cassette might provide yet another tool for detailed regional and developmental expression analysis using colorimetric beta-galactosidase substrates.

Given the previously described role in the nervous system, we have focused our efforts on addressing the neurobehavioral consequences of CPD1 loss. While the mice do not exhibit deficits on the accelerating rotarod, they do show deficits on the balance beam, a more stringent assay to detect gait disorders in mice. These subtle motor deficits are also consistent with the clasping phenotype that these mice display. It is possible that the minor phenotype might reflect differences in mouse background. However, given that we have back crossed the mice for five generations (onto the C57Bl6 background), we think that this scenario is unlikely.

The minimal phenotype of CPD1 loss, LANP loss (described in an earlier study; [3]) or indeed loss of both proteins as exemplified by the double knock-out mice warrants further comment. We take these results to suggest one of several possibilities: The first possibility is that ANP homologues such as LANP and CPD1 play distinct roles that modulate cellular functions, but are not essential for life. Depleting them either individually or in a combined manner would thus lead to minimal phenotypes, perhaps exaggerated in pathological situations— in keeping with the putative role of ANP proteins in neurodegeneration and cancer. Alternatively, given the evolutionarily conserved nature of these proteins and the observation that they overlap in expression patterns and functional properties of these proteins, we suspect that as a group ANP proteins are indeed essential for vital functions. We speculate these functions will only become apparent by generating sequential knock-outs of other family member(s) as well. This scenario is reminiscent of other evolutionary conserved family members (such as the neuroligin family), where multiple knock-outs are required [22]. The next logical choice in a sequential gene targeting approach would be to target the close LANP and CPD1 homologue APRIL (A Protein Rich in Leucines; aka SSP-29, PAL31 and ANP32B NCBI Reference Sequence: NP_570959.1 with 272 amino acids) [23]. We should point out that two ANP proteins that are smaller in size—ANP32c and ANP32d (234 amino acids and 131 amino acids respectively) —have been speculated to play a role in tumorigenesis [24], but it is not clear if they are expressed in neurons. Based on their absence in expressed sequence tag databases and the fact that their genes are intronless they might well be pseudogenes expressed in pathological situations.

The LANP and now CPD1 null mice that we have developed provide an invaluable resource. For instance, they can be used to perform cell-based assays to test for ANP ascribed regulatory functions underlying: intracellular signaling, mRNA stability and transport; cytoskeletal dynamics/vesicle transport (via effects on microtubule associated proteins), cell death (via effects on apoptosis pathways [13], and finally, chromatin modulation and gene expression (via effects on histone acetylation) [4], [25], [26], [27]). Moreover, both LANP and CPD1 have been proposed to play an important role in tumorigenesis [17], [24], [28] and neurodegeneration [2], [8]. In these contexts as well, our mice should prove handy. For instance, the cells of these mice could be used to study pathways relevant to tumorigenesis and neurodegeneration. More importantly, by mating to CPD1 knockout mice to various tumor and neurodegenerative models a deeper insight into disease regulation and progression can be achieved.

## Supporting Information

Text S1 Supplementary text
(0.03 MB DOC)

Figure S1 Genotyping of CPD1 null mice: A. Schematic of CPD1 deletion strategy depicts location of β-geo (β-galactosidase+ neomycin R) cassette between Exons 3 and 4 (not drawn to scale). B. Genotyping was performed on DNA extracted from tail clips of mice using primers 5F, 6R, Neo F, and Neo R yielding 580 bp band (WT) and 300 bp band (KO). CPD+/− mice displayed both bands corresponding to each allele.
(0.64 MB TIF)

Figure S2 Depletion of CPD1 upon RNA interference. A. Western blot analysis of CPD1 expression from PC12 cell lysates transfected with mock, control, and siRNA for CPD1. Actin is used as a loading control. B. Immunostaining of PC12 cells transfected with mock, control, and siRNA for CPD1with CPD1 antibody. Scale bar = 50 µm.
(1.91 MB TIF)

Movie S1 Video of CPD1 −/− mouse shows clasping behavior. When held from their tail, CPD1 null mice tend to clasp their hind limbs towards their belly for durations ranging from 1–3 seconds.
(3.35 MB MPG)

Movie S2 Video of CPD1 +/+ mouse as a wild type control. When held from their tail, wild type mice tend to position their hind limbs and paws outwards and away from their belly.
(3.55 MB MPG)

## References

[1] Cummings CJ, Sun Y, Opal P, Antalffy B, Mestril R (2001). Over-expression of inducible HSP70 chaperone suppresses neuropathology and improves motor function in SCA1 mice.. Hum Mol Genet.

[2] Cvetanovic M, Rooney RJ, Garcia JJ, Toporovskaya N, Zoghbi HY (2007). The role of LANP and ataxin 1 in E4F-mediated transcriptional repression.. EMBO Rep.

[3] Opal P, Garcia JJ, McCall AE, Xu B, Weeber EJ (2004). Generation and Characterization of LANP/pp32 Null Mice.. Mol Cell Biol.

[4] Opal P, Garcia JJ, Propst F, Matilla A, Orr HT (2003). Mapmodulin/leucine-rich acidic nuclear protein binds the light chain of microtubule-associated protein 1B and modulates neuritogenesis.. J Biol Chem.

[5] Matilla A, Radrizzani M (2005). The Anp32 family of proteins containing leucine-rich repeats.. Cerebellum.

[6] Matsuoka K, Taoka M, Satozawa N, Nakayama H, Ichimura T (1994). A nuclear factor containing the leucine-rich repeats expresses in murine cerebellar neurons.. Proc Natl Acad Sci USA.

[7] Radrizzani M, Vila-Ortiz G, Cafferata EG, Di Tella MC, Gonzalez-Guerrico A (2001). Differential expression of CPD1 during postnatal development in the mouse cerebellum.. Brain Res.

[8] Matilla A, Koshy BT, Cummings CJ, Isobe T, Orr HT (1997). The cerebellar leucine-rich acidic nuclear protein interacts with ataxin-1.. Nature.

[9] Kular RK, Cvetanovic M, Siferd S, Kini AR, Opal P (2009). Neuronal Differentiation Is Regulated by Leucine-rich Acidic Nuclear Protein (LANP), a Member of the Inhibitor of Histone Acetyltransferase Complex.. J Biol Chem.

[10] Li M, Makkinje A, Damuni Z (1996). Molecular identification of I1PP2A, a novel potent heat-stable inhibitor protein of protein phosphatase 2A.. Biochemistry.

[11] Costanzo RV, Vila-Ortiz GJ, Perandones C, Carminatti H, Matilla A (2006). Anp32e/Cpd1 regulates protein phosphatase 2A activity at synapses during synaptogenesis.. Eur J Neurosci.

[12] Vaesen M, Barnikol-Watanabe S, Gotz H, Awni LA, Cole T (1994). Purification and characterization of two putative HLA class II associated proteins: PHAPI and PHAPII.. Biol Chem Hoppe Seyler.

[13] Jiang X, Kim HE, Shu H, Zhao Y, Zhang H (2003). Distinctive roles of PHAP proteins and prothymosin-alpha in a death regulatory pathway.. Science.

[14] Fan Z, Beresford PJ, Oh DY, Zhang D, Lieberman J (2003). Tumor Suppressor NM23-H1 Is a Granzyme A-Activated DNase during CTL-Mediated Apoptosis, and the Nucleosome Assembly Protein SET Is Its Inhibitor.. Cell.

[15] Brennan CM, Gallouzi IE, Steitz JA (2000). Protein ligands to HuR modulate its interaction with target mRNAs in vivo.. J Cell Biol.

[16] Chen TH, Brody JR, Romantsev FE, Yu JG, Kayler AE (1996). Structure of pp32, an acidic nuclear protein which inhibits oncogene-induced formation of transformed foci.. Mol Biol Cell.

[17] Bai J, Brody JR, Kadkol SS, Pasternack GR (2001). Tumor suppression and potentiation by manipulation of pp32 expression.. Oncogene.

[18] Brody JR, Witkiewicz A, Williams TK, Kadkol SS, Cozzitorto J (2007). Reduction of pp32 expression in poorly differentiated pancreatic ductal adenocarcinomas and intraductal papillary mucinous neoplasms with moderate dysplasia.. Mod Pathol.

[19] Stryke D, Kawamoto M, Huang CC, Johns SJ, King LA (2003). BayGenomics: a resource of insertional mutations in mouse embryonic stem cells.. Nucleic Acids Res.

[20] Mutai H, Toyoshima Y, Sun W, Hattori N, Tanaka S (2000). PAL31, a novel nuclear protein, expressed in the developing brain.. Biochem Biophys Res Commun.

[21] Heng MY, Tallaksen-Greene SJ, Detloff PJ, Albin RL (2007). Longitudinal evaluation of the Hdh(CAG)150 knock-in murine model of Huntington's disease.. J Neurosci.

[22] Varoqueaux F, Aramuni G, Rawson RL, Mohrmann R, Missler M (2006). Neuroligins determine synapse maturation and function.. Neuron.

[23] Mencinger M, Panagopoulos I, Contreras JA, Mitelman F, Aman P (1998). Expression analysis and chromosomal mapping of a novel human gene, APRIL, encoding an acidic protein rich in leucines.. Biochim Biophys Acta.

[24] Kadkol SS, Brody JR, Pevsner J, Bai J, Pasternack GR (1999). Modulation of oncogenic potential by alternative gene use in human prostate cancer.. Nat Med.

[25] Ulitzur N, Rancaño C, Pfeffer SR (1997). Biochemical characterization of mapmodulin, a protein that binds microtubule-associated proteins.. The J Biol Chem.

[26] Ulitzur N, Humbert M, Pfeffer SR (1997). Mapmodulin: a possible modulator of the interaction of microtubule- associated proteins with microtubules.. Proc Natl Acad Sci U S A.

[27] Itin C, Ulitzur N, Muhlbauer B, Pfeffer SR (1999). Mapmodulin, cytoplasmic dynein, and microtubules enhance the transport of mannose 6-phosphate receptors from endosomes to the trans-golgi network.. Mol Biol Cell.

[28] Kadkol SS, Brody JR, Epstein JI, Kuhajda FP, Pasternack GR (1998). Novel nuclear phosphoprotein pp32 is highly expressed in intermediate- and high-grade prostate cancer.. Prostate.

