# Supplementary material for: Cpd-1 Null Mice Display a Subtle Neurological Phenotype
Source: PLoS One. 2010 Sep 9;5(9):e12649. doi: 10.1371/journal.pone.0012649 (PMC2936576; doi:10.1371/journal.pone.0012649)
Supplement: Text S1 — Supplementary text (0.03 MB DOC) [file pone.0012649.s001.doc]

**Supplementary methods:**

**Genotyping:** DNA extracted from tail clips of the mice was genotyped using primer 5F: CTGTGCGTGTTGTTTTCTG, 6R: GTCATCCTCCTCCTCCGAGT, Neo F: CTTGGGTGGAGAGGCTATTC, and Neo R:AGGTGAGATGACAGGAGATC. Neomycin specific marker serves as a DNA quality control for Cpd1-/- mice, which will not amplify the 5F to 6R band due to the size of the cassette. The resultant 4 primer genotyping assay yields 2 bands. The first is 580bp and is specific to the wild type allele. The second is 300bp and is specific to the Neomycin portion of the cassette only found in mutants.

**RNA interference:** ON-TARGET plus siRNA targeting CPD1 (target sequence: UCACAUACUUGGACGGAUU) was obtained from Dharmacon. PC12 cells were plated at the cell density of 200, 000 cells/well of 6-well plate and transfected with control siRNA and siRNA targeting CPD1, along with mock transfected wells using lipofectamine (Invitrogen) according to the manufacturer’s instructions. A set of two transfections on two consecutive days was used for optimal transfection efficiency. A day after the second transfection , the cells were trypsinized and plated on coverslips at low density (50,000 cells/well of 24 well plate) and in 6-well plates at high density (250,000 cells/well of 6-well plate) for protein analysis. 24 hours later protein lysates were obtained for western blot analysis. The cells were fixed and stained with CPD1 antibody and fluorescently labeled secondary antibody (Jackson Immunochemicals) for immunofluorescence. Microscopy was performed using a Zeiss Axiovert microscope. Images were processed with Adobe Photoshop 7.0 software.
