# Supplementary figures and images for: Cpd-1 Null Mice Display a Subtle Neurological Phenotype
Source: PLoS One. 2010 Sep 9;5(9):e12649. doi: 10.1371/journal.pone.0012649 (PMC2936576; doi:10.1371/journal.pone.0012649)

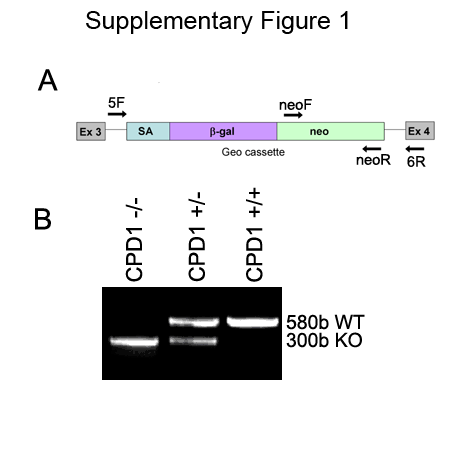

Supplement: Figure S1 — Genotyping of CPD1 null mice: A. Schematic of CPD1 deletion strategy depicts location of β-geo (β-galactosidase+ neomycin R) cassette between Exons 3 and 4 (not drawn to scale). B. Genotyping was performed on DNA extracted from tail clips of mice using primers 5F, 6R, Neo F, and Neo R yielding 580 bp band (WT) and 300 bp band (KO). CPD+/− mice displayed both bands corresponding to each allele. (0.64 MB TIF) [file pone.0012649.s002.tif]

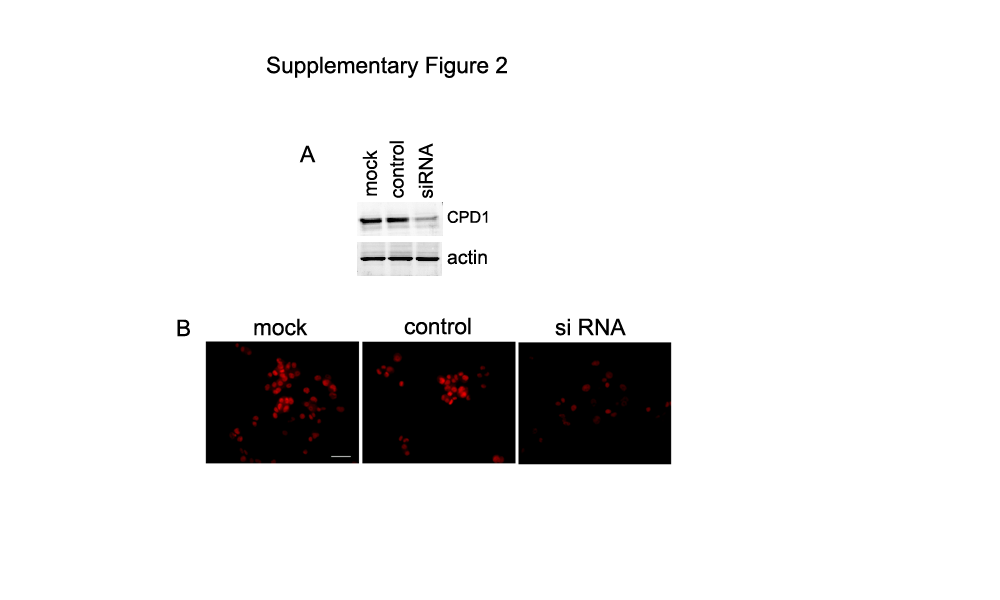

Supplement: Figure S2 — Depletion of CPD1 upon RNA interference. A. Western blot analysis of CPD1 expression from PC12 cell lysates transfected with mock, control, and siRNA for CPD1. Actin is used as a loading control. B. Immunostaining of PC12 cells transfected with mock, control, and siRNA for CPD1with CPD1 antibody. Scale bar = 50 µm. (1.91 MB TIF) [file pone.0012649.s003.tif]
